# Ex vivo ^18^F-fluoride uptake and hydroxyapatite deposition in human coronary atherosclerosis

**DOI:** 10.1038/s41598-020-77391-6

**Published:** 2020-11-19

**Authors:** Alastair J. Moss, Alisia M. Sim, Philip D. Adamson, Michael A. Seidman, Jack P. M. Andrews, Mhairi K. Doris, Anoop S. V. Shah, Ralph BouHaidar, Carlos J. Alcaide-Corral, Michelle C. Williams, Jonathon A. Leipsic, Marc R. Dweck, Vicky E. MacRae, David E. Newby, Adriana A. S. Tavares, Stephanie L. Sellers

**Affiliations:** 1 BHF Centre for Cardiovascular Science, Chancellor’s Building, University of Edinburgh, 49 Little France Crescent, Edinburgh, EH16 4SB UK; 2 British Heart Foundation Cardiovascular Research Centre, University of Leicester, Leicester, UK; 3 EaStCHEM School of Chemistry, University of Edinburgh, Edinburgh, UK; 4 Christchurch Heart Institute, University of Otago, Christchurch, New Zealand; 5 Department of Pathology, St Paul’s Hospital and University of British Columbia, Vancouver, Canada; 6 Department of Radiology and Centre for Heart Lung Innovation, St Paul’s Hospital and University of British Columbia, Vancouver, Canada; 7 The Roslin Institute and Royal (Dick) School of Veterinary Studies, University of Edinburgh, Edinburgh, UK

**Keywords:** Biomarkers, Cardiology, Molecular medicine

## Abstract

Early microcalcification is a feature of coronary plaques with an increased propensity to rupture and to cause acute coronary syndromes. In this ex vivo imaging study of coronary artery specimens, the non-invasive imaging radiotracer, ^18^F-fluoride, was highly selective for hydroxyapatite deposition in atherosclerotic coronary plaque. Specifically, coronary ^18^F-fluoride uptake had a high signal to noise ratio compared with surrounding myocardium that makes it feasible to identify coronary mineralisation activity. Areas of ^18^F-fluoride uptake are associated with osteopontin, an inflammation-associated glycophosphoprotein that mediates tissue mineralisation, and Runt-related transcription factor 2, a nuclear protein involved in osteoblastic differentiation. These results suggest that ^18^F-fluoride is a non-invasive imaging biomarker of active coronary atherosclerotic mineralisation.

## Introduction

Coronary atherosclerosis is an inflammatory disease that results in the formation of intimal plaque with an increased propensity to rupture. Microscopic calcification is a key feature of ruptured atherosclerotic plaques and the identification of coronary microcalcification is closely linked to coronary thrombotic events^1^. However, the in vivo mechanisms governing the accumulation of early microscopic calcification within the coronary vasculature are poorly understood. Pre-clinical studies have proposed atherosclerotic inflammation to be an initiator of plaque calcification through the extrusion and response to calcifying extracellular vesicles^2,3^. Additionally, in vitro models of intimal plaque microcalcification have demonstrated that spherical or ellipsoidal micro-calcifying vesicles aggregate within plaques and coalesce to form larger plates of macrocalcification^3^. Whilst the transition from microcalcification to macrocalcification in the vast majority of plaques is thought to confer stability, the presence of micro-calcifying vesicles in the tunica intima has the potential to reduce the structural integrity of thin-capped fibroatheroma, resulting in plaque rupture^4,5^.

Recently, studies have demonstrated that increased ^18^F-sodium fluoride (^18^F-fluoride) positron emission tomography (PET) uptake is observed in culprit plaques following myocardial infarction and in plaques with multiple adverse features in patients with stable disease^1,6–8^. ^18^F-Fluoride preferentially binds to exposed hydroxyl groups on the surface of nanocystalline hydroxyapatite. We have previously demonstrated that the signal intensity of ^18^F-fluoride in carotid endarterectomy specimens increases as the size of the calcifications decrease, such that ^18^F-fluoride is an imaging biomarker of unbound microscopic calcification^9,10^. However, there are important differences between carotid and coronary atherosclerotic plaque progression, predominantly attributed to plaque composition. Compared with carotid plaques, vulnerable coronary plaques are more prone to rupture owing to thinner fibrous caps (< 65 µm versus < 200 µm) and a reduction in smooth muscle cells in the tunica media^11^. To address these differences in pathophysiology and to fully understand the mechanisms of ^18^F-fluoride binding in coronary atherosclerotic plaque, direct histological examination of coronary artery tissue is warranted^12^. In this study, we performed an ex vivo histological validation of ^18^F-fluoride binding to calcium derivatives and osteogenic proteins involved in human coronary atherosclerotic calcification.

## Results

### Selectivity of ^18^F-fluoride for hydroxyapatite

**Figure 1:**
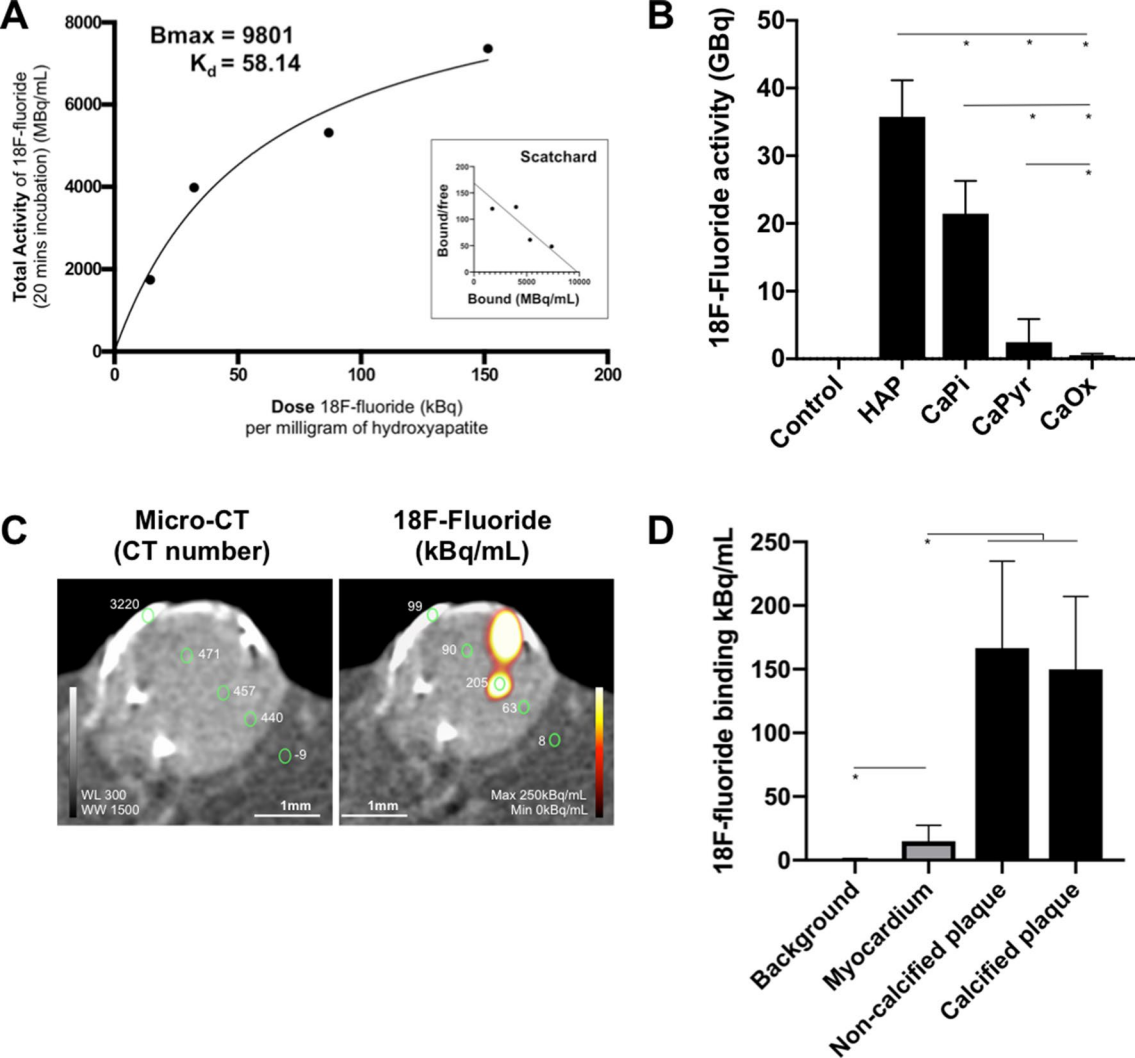
^18^F-Fluoride binding in ex vivo coronary artery specimens. (**A**) Total activity of ^18^F-fluoride (MBq/mL) following 20 min incubation in hydroxyapatite plotted against dose of ^18^F-fluoride (kBq) per milligram of hydroxyapatite. Scatchard plot of bound/free versus bound ^18^F-fluoride (inset). (**B**) Hydroxyapatite and other calcium-derived minerals were incubated with ^18^F-fluoride for 20 min after which unbound supernatant was removed and the solid particles were analysed by micro positron emission tomography. Data shown is from at least three replicates and shown as the mean ± standard deviation. (**C**) Micro-computed tomography defined regions of myocardium (CT number < 100) from coronary plaque (CT number > 300). Calcification had high CT attenuation on micro-computed tomography (CT number > 1000). Quantitative ^18^F-fluoride positron tomography was analysed on hybrid images. (**D**) Non-specific binding in the surrounding myocardium was higher than background activity (median 14.9 [interquartile range 9.6–27.4] versus 0.30 [interquartile range, 0.09–1.3] kBq/mL respectively, p < 0.0001). Signal in coronary artery segments was tenfold higher (p < 0.0001) than myocardium (non-calcified plaque median 158.1 [interquartile range, 121.7–234.8] kBq/mL and calcified plaque median 149.8 [interquartile range, 85.3–207.3] kBq/mL). *CaPi* calcium bisphosphate, *CaPyr* calcium pyrophosphate, *CaOx* calcium oxalate, *HAP* hydroxyapatite, *WL* window level, *WW* window width. *Indicates p < 0.0001

^18^F-Fluoride had favourable equilibrium kinetics (Bmax 9.8 kBq/mL, K_d_ 58.14 kBq) for hydroxyapatite with binding equilibrium occurring within 20 min (Fig. 1A). To achieve target saturation, 100 kBq of ^18^F-fluoride was used for subsequent ex vivo experiments. There was high selectivity of ^18^F-fluoride for hydroxyapatite in comparison with other calcium-phosphate derivatives (calcium bisphosphate, p < 0.01; calcium pyrophosphate p < 0.001) (Fig. 1B). Very low activity was observed in calcium oxalate samples incubated with ^18^F-fluoride (p < 0.001).

### Study population

Between 2016 and 2017, coronary artery samples were obtained at autopsy from 13 victims of sudden death. The majority were male (n = 10, 76.9%) with a median age of 51 (range 40–71) years. Ten deaths (76.9%) were adjudicated as related to ischemic heart disease, one death was attributed to haemopericardium from thoracic aortic dissection and two deaths were related to non-cardiac causes (suffocation and alcohol toxicity). None of the coronary artery specimens represented culprit or ruptured plaque directly related to the sudden death.

### ^18^F-Fluoride co-localisation with hydroxyapatite

**Figure 2:**
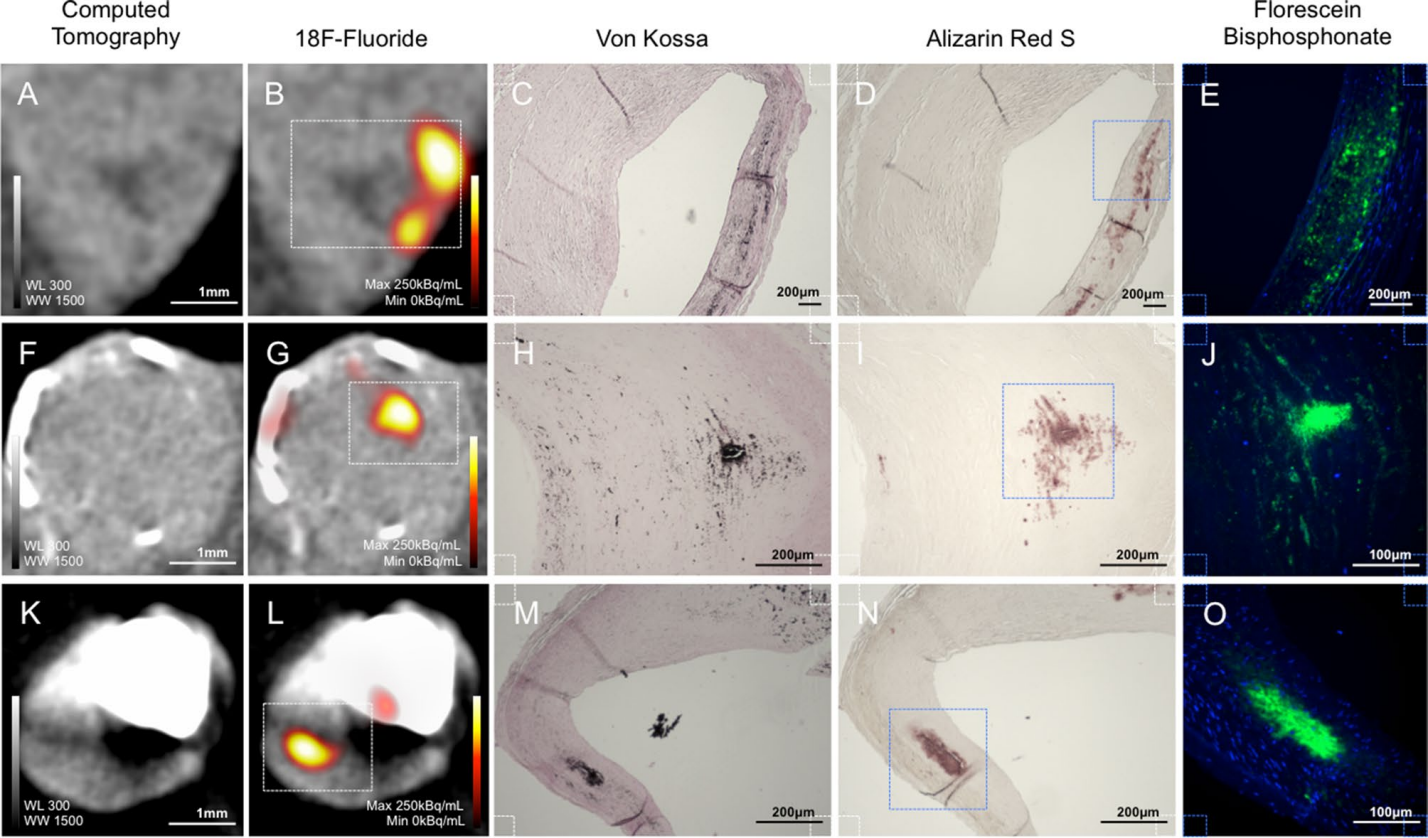
^18^F-Fluoride, coronary microcalcification and hydroxyapatite deposition. Coronary artery specimens had no macrocalcification detected by micro-computed tomography (**A**) and macrocalcification in a circumferential pattern (**F**) or large calcified nodules (**K**). High-intensity ^18^F-Fluoride binding occurred in regions remote from these regions of macrocalcification observed on micro-computed tomography in the intimal layer of fibroatheromatous plaques (**B**,**G**,**L**). Low-intensity ^18^F-fluoride activity was noted on the exposed surface of macrocalcified deposits (**G**,**L**). Magnified histological analysis (white dotted region) demonstrated co-localisation with evidence of tissue mineralisation using Von Kossa staining (**C**,**H**,**M**), microcalcification using Alizarin Red S staining (**D**,**I**,**N**) and hydroxyapatite deposition using a hydroxyapatite-specific bisphosphonate probe (blue dotted region), fluorescein-bisphosphonate [green] (**E**,**J**,**O**). *WL* window level, *WW* window width.

Within tissue sections from the 13 patients, 32 coronary artery plaques were identified using high-resolution micro-computed tomography. Specifically, the morphology and architecture combined with the CT attenuation number of the tissue differentiated coronary artery plaques (CT number > 300) from the surrounding myocardium (CT number < 100) (Fig. 1C). ^18^F-Fluoride binding was observed in plaques both with (n = 19) and without (n = 13) areas of macroscopic calcification as determined on micro-CT. Total plaque ^18^F-fluoride binding (median 157.5 [IQR 103.9–216.9] kBq/mL) was more than tenfold greater than non-specific binding in the surrounding myocardium (median 14.9 [IQR 9.6–27.4] kBq/mL, p < 0.0001) and more than 500-fold greater than background regions (median 0.3 [IQR 0.09–1.3] kBq/mL, p < 0.0001) (Fig. 1D). ^18^F-Fluoride activity in coronary plaques without macroscopic calcification (median 158.1 [IQR 121.7–234.8] kBq/mL) was higher than plaque with macrocalcification (median 149.8 [IQR 85.3–207.3] kBq/mL; p = 0.0469). In plaques with no observable macrocalcification on micro-computed tomography, nanocrystalline calcification was detected using high-intensity ^18^F-fluoride in focal regions co-localised to fibroatheromatous plaques (Fig. 2A–D). In plaques with macrocalcification, high-intensity ^18^F-fluoride uptake was observed in distinct regions remote from larger macrocalcific deposits identified on micro-computed tomography (Fig. 2F,G,K,L). Additional low-intensity ^18^F-fluoride uptake was observed on the exposed surface of macrocalcification (Fig. 2G,L). In the high-intensity regions, ^18^F-fluoride colocalised with histological evidence of microcalcification shown by Von Kossa and Alizarin Red S staining (Fig. 2H,I and M,N respectively). Moreover the microcalcification observed in areas of ^18^F-fluoride binding was specifically classified as hydroxyapatite using a highly selective immunofluorescence probe, fluorescein-bisphosphonate (Fig. 2E,J,O).

### Raman spectroscopy and ^18^F-fluoride intensity

**Figure 3:**
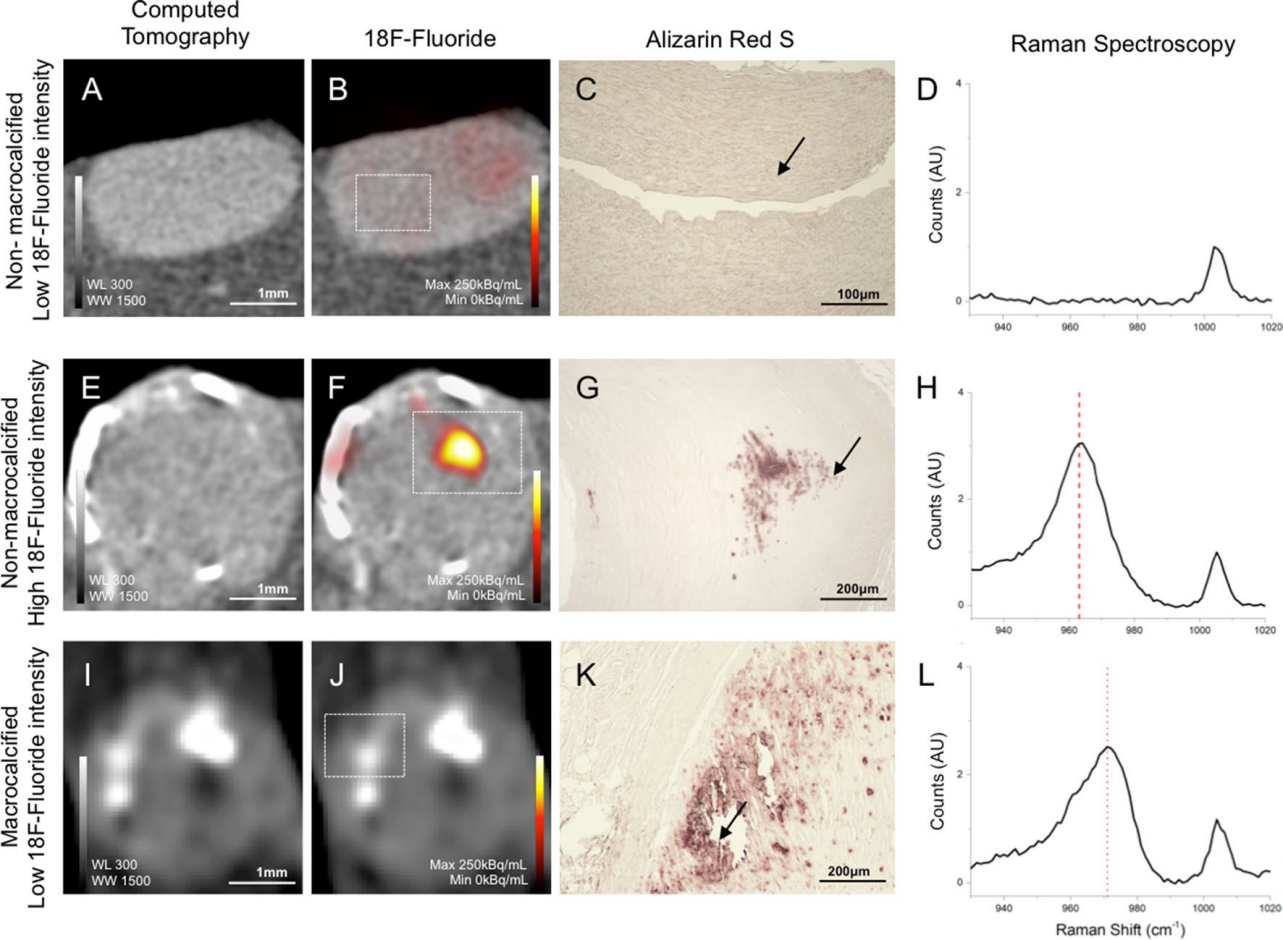
^18^F-Fluoride with Raman spectroscopy for hydroxyapatite and whitelockite. Coronary artery specimens with high-intensity ^18^F-fluoride binding and low-intensity ^18^F-fluoride binding underwent Raman spectroscopy analysis to differentiate mineral composition (n = 8). Non-macrocalcified specimens with low-intensity ^18^F-fluoride intensity (control, n = 1) had a single spectra peak at 1004 cm^−1^ corresponding to phenylalanine (**A**–**D**). Focal regions with no macrocalcification and high-intensity ^18^F-fluoride intensity (n = 3) had a Raman spectra peak at 963 cm^−1^, hydroxyapatite (**E**–**H**). Macrocalcified regions with low-intensity ^18^F-fluoride intensity had a Raman spectra peaks at 975 cm^−1^, whitlockite (**I**–**L**). Raman Shift: Hydroxyapatite, 963 cm^−1^, Whitlockite, 973 cm^−1^, Phenylalanine, 1004 cm^−1^. *WL* window level, *WW* window width.

Raman spectroscopy imaging of selected regions with high and low-intensity ^18^F-fluoride binding was performed in eight samples (Fig. 3A–L). The characteristic cell Raman signal was observed in all these samples at 1004 cm^−1^ corresponding to phenylalanine. In a control sample with no ^18^F-fluoride signal or calcification on Alizarin Red S staining (Fig. 3A–C), no Raman signal was observed in the range between 940 and 1000 cm^−1^ (Fig. 3D). Samples containing microcalcification identified by high-intensity ^18^F-fluoride (157 kBq/mL) (Fig. 3E–G) had a Raman signal at 963 cm^−1^ corresponding to hydroxyapatite (Fig. 3H). In macrocalcified specimens with low-intensity ^18^F-fluoride (30 kBq/mL) (Fig. 3I–K), Raman signal with an asymmetrical v1 stretching band appeared at 973 cm^−1^ corresponding to whitlockite (Fig. 3L). As there was an asymmetrical peak to signal in these macrocalcified specimens, further analysis of the area under the whitlockite peak revealed two overlapping peaks attributable to hydroxyapatite and whitlockite at a ratio of 30:70.

### ^18^F-Fluoride co-localisation with markers of osteogenic activity in coronary arteries

**Figure 4:**
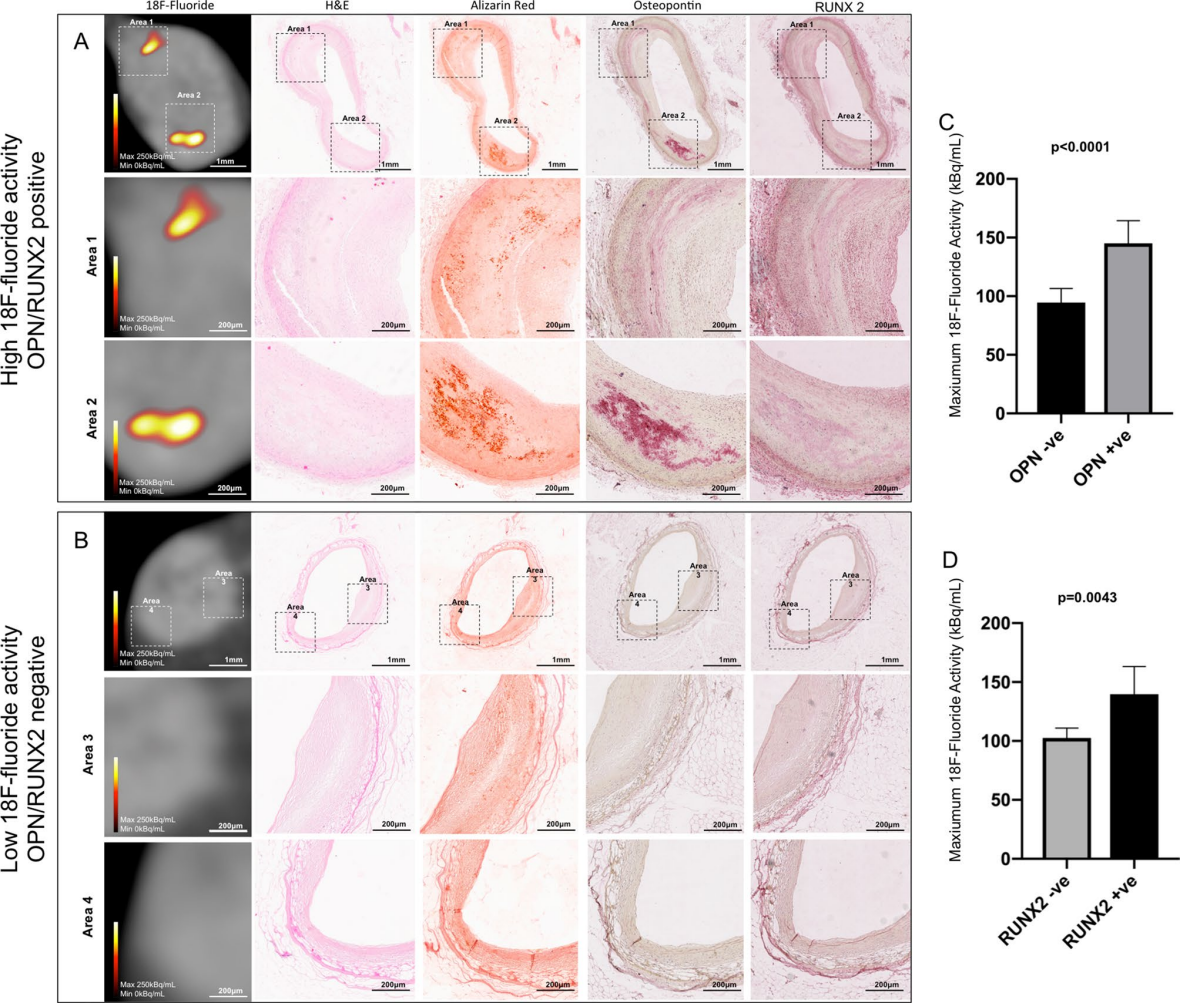
^18^F-Fluoride and transcription factors associated with atherosclerotic mineralisation. Fibroatheromatous plaques with high ^18^F-fluoride activity had an abundance of microcalcification (Alizarin Red S) (**A**). The distribution of the biomineralisation glycophosphoprotein osteopontin and the nuclear factor of osteoblastic differentiation Runt-related transcription factor 2 colocalised with microcalcification in the intimal layer (**A**). In regions without high-intensity ^18^F-fluoride activity no deposition of osteopontin or Runt-related transcription factor 2 was seen (**B**). High-intensity ^18^F-fluoride activity was associated with markers of inflammatory mediated mineralisation (Osteopontin: positive 145.0 kBq/mL versus negative 94.5 kBq/mL, difference in means 50.5 [95% confidence interval 28.1–72.9] kBq/mL, p < 0.0001 (**C**); Runt-related transcription factor 2: positive 139.8 kBq/mL versus negative 102.6 kBq/mL, difference in means 37.2 [95% confidence interval 12.6–61.8] kBq/mL, p = 0.0043) (**D**). OPN, Osteopontin, RUNX-2, Runt-related transcription factor 2.

Detailed analysis of coronary artery specimens revealed that ^18^F-fluoride binding was predominantly observed within the tunica intima in areas of plaque formation (Fig. 4A). Little or no binding was observed in the tunica media. In coronary atherosclerotic plaques without macrocalcification, there was no intense staining for alkaline phosphatase (weakly positive) despite high-intensity ^18^F-fluoride activity. However, these regions of high-intensity ^18^F-fluoride co-localised with the increased expression of osteopontin (Fig. 4A). Osteopontin deposition was not observed in regions without high ^18^F-fluoride activity (Fig. 4B). Osteopontin-positive plaques had higher ^18^F-fluoride activity compared to those without (mean 145 versus 94.5 kBq/mL respectively: difference in means 50.5 [95% confidence interval 28.1–72.9] kBq/mL, p < 0.0001) (Fig. 4C). Similarly, Runt-related transcription factor 2 (Runx-2) positive plaques had higher ^18^F-fluoride activity compared to those without (mean 139.8 versus 102.6 kBq/mL respectively; difference in means 37.2 [95% confidence interval 12.6–61.8] kBq/mL, p = 0.0043) (Fig. 4D). Staining for transforming growth factor beta 1 and caspase 3 was evident in these plaques, particularly in regions of macrocalcification. However, ^18^F-fluoride activity did not correlate with increased staining for transforming growth factor beta 1 (TGFβ1, p = 0.1042), wingless/integrated 3a (WNT3A, p = 0.8732) or caspase 3 (p = 0.5476).

## Discussion

In this ex vivo imaging study of coronary atherosclerosis, we have demonstrated for the first time that ^18^F-fluoride is a selective marker of intimal hydroxyapatite deposition in human coronary atherosclerotic plaques. Similar to other disease states it preferentially binds in areas of coronary microcalcification rather than macrocalcification. ^18^F-Fluoride has a high affinity for hydroxyapatite, which has a higher surface area for binding in regions of microcalcification compared with larger macrocalcified deposits. Importantly, high ^18^F-fluoride signal co-localises with the distribution of osteopontin and Runx-2, established markers of early calcification activity and adverse plaque formation. This histological validation supports the use of ^18^F-fluoride positron emission tomography as a marker of developing microcalcification and plaque activity in patients with coronary artery disease.

Whilst there has been histological confirmation of ^18^F-fluoride binding in carotid atheroma, studies demonstrating increased ^18^F-fluoride activity in the coronary arteries^1,7^ have been called into question due to the limited spatial resolution of clinical positron emission tomography, with some investigators questioning whether ^18^F-fluoride binding actually occurs in coronary arteries^14^. In this regard, the confirmation of high-intensity ^18^F-fluoride binding in the intimal layer of coronary plaques compared to background and adjacent myocardium is of considerable importance. We have also confirmed that ^18^F-fluoride binding occurs in plaques both with and without macroscopic calcium observed on CT, and that binding appears to occur preferentially in regions of developing microcalcification. These findings are consistent with previous observations in coronary artery disease as well the data in carotid atheroma and other cardiovascular disease states^9,10,15–17^.

Of particular interest is the potential for ^18^F-fluoride to discriminate hydroxyapatite deposition above other calcium derivatives in regions of active mineralisation^18^. Of the many calcium derivatives, nanocrystalline hydroxyapatite is the central component of microcalcification in atherosclerotic coronary plaques^18^. We have here confirmed the preferential binding of ^18^F-fluoride for microcalcification and for hydroxyapatite based upon the binding of a specific optical probe and Raman spectroscopy. At later stages in the calcification process, other calcium derivatives, such as whitlockite, become more abundant in calcified vascular tissue, particularly within large vessel atherosclerosis where there is often a high whitlockite to hydroxyapatite ratio^19^. The phase transformation of hydroxyapatite to whitlockite may occur in the hypoxic or acidic conditions within necrotic cores where magnesium ions are incorporated onto the surface and prevent further growth of hydroxyapatite crystals^20^. Traditionally surface area effects have been used to explain the preferential binding of ^18^F-fluoride for microcalcification. However, the specificity of ^18^F-fluoride for hydroxyapatite provides an additional explanation for why high ^18^F-fluoride activity is not observed in areas macrocalcification and why it provides different information to CT^9^.

We also observed a close relationship between the coronary ^18^F-fluoride signal and both osteopontin and Runx-2 expression, established markers of early calcification activity and adverse coronary plaque. High concentrations of osteopontin accumulate in coronary atheroma exposed to hypoxia and endothelial injury^21^. Of note, inflammatory signalling within metabolically active coronary plaques stimulates macrophage-derived foam cells to express high levels of osteopontin^22^. In comparison, low levels of osteopontin mRNA are found in vascular smooth muscle cells often regarded as the cell type responsible for initiating plaque calcification^23^. Importantly, from a clinical perspective, high plasma osteopontin levels are associated with adverse clinical events in patients with both stable and unstable coronary artery disease^24,25^. Combined with high-sensitivity C reactive protein, osteopontin had a two-fold increased risk of recurrent myocardial infarction in patients presenting with ST elevation myocardial infarction^25^. The role of osteopontin in mediating plaque activity is noted by the beneficial effect of statins in reducing osteopontin levels and thereby reduce the risk of plaque rupture^26^. The relationship between ^18^F-fluoride uptake and osteopontin therefore supports its role as a marker of early calcification activity and adverse plaque formation. However, ultimately data are required to investigate whether ^18^F-fluoride predicts future myocardial infarction and therefore might provide important clinical information. In this regard, to determine whether coronary ^18^F-fluoride has clinical utility, prognostic observation studies in patients with recent myocardial infarction are ongoing (NCT02278211).

Co-localisation of ^18^F-fluoride uptake to specific coronary atheromatous plaques presents some challenges when conducting clinical positron emission tomography and computed tomography coronary angiography in patients with coronary artery disease. The small calibre of the coronary arteries leads to partial volume averaging, and the near continuous motion from cardiac and respiratory cycles can limit signal localisation to specific regions of the coronary circulation. Some of these issues can be improved by use of beta-blockade, motion correction and advanced image analysis techniques^27^. The present study is therefore important to reaffirm that ^18^F-fluoride is binding to individual advanced human coronary atheromatous plaques as well as identify the components to which it binds. The emerging clinical application of ^18^F-fluoride positron emission tomography and computed tomography coronary angiography is able to provide clinicians with a tool to monitor disease activity and identify individuals at increased risk of future coronary events^28^. Since the probability of an individual ruptured plaque causing an acute coronary event is low, ^18^F-fluoride positron emission tomography and computed tomography coronary angiography is best utilised to detect overall coronary atherosclerotic disease activity in vulnerable patients rather than localising specifically to a single vulnerable plaque^29^.

There are some limitations to this study. Legislation regarding the regulation of tissue in victims of sudden death meant that only left main and proximal left anterior descending coronary artery specimens could be obtained for detailed research analysis. These specimens did not include sections of culprit coronary plaque rupture with thrombus formation and therefore extrapolation of these findings to ruptured atherosclerotic plaques cannot be made. Additionally, specimen preparation and handling between different imaging modalities may have resulted in alterations in the orientation and alignment of the datasets. Ante-mortem demographics regarding risk factors such as diabetes mellitus and renal disease which influence atherosclerotic calcification were unavailable. However, the majority of cases in this study were adjudicated by a forensic pathologist who determined a cause of death attributed to ischemic heart disease independent from the study investigators. This provides further evidence of the high prevalence of ^18^F-fluoride binding in coronary arteries in a high-risk cohort. Although the significance of the high frequency of sudden cardiac death (77%) in this study population is uncertain, further studies exploring the utility of coronary ^18^F-fluoride imaging in victims of sudden death are worth pursuing.

## Conclusions

In this ex vivo study of coronary atherosclerotic plaques, ^18^F-fluoride binding was highly selective for unbound hydroxyapatite deposition. High ^18^F-fluoride intensity was associated with intimal microcalcification in regions of osteopontin and Runx-2 expression. This study provides further evidence to support the use of ^18^F-fluoride positron emission tomography as a marker of plaque vulnerability in patients with coronary artery disease.

## Methods

### Saturation binding assays to quantify ^18^F-fluoride binding kinetics and selectivity to hydroxyapatite

Saturation radioligand binding experiments to determine the number of binding sites (Bmax) and the dissociation constant (K_d_) of ^18^F-fluoride were undertaken using nanocrystalline hydroxyapatite phantoms prior to performing ex vivo imaging. Five-milligram vials of hydroxyapatite were incubated with ^18^F-fluoride (110, 230, 470 or 700 kBq/mL) for 20 min. The supernatant fraction was then removed and hydroxyapatite was twice washed in 10 mL 0.9% sodium chloride for 5 min to remove unbound ^18^F-fluoride. Hydroxyapatite phantoms were scanned using high-resolution micro-PET (1:5 coincidence mode) and computed tomography (CT) with semi-circular full trajectory, maximum field of view, 480 projections, 50 kVp, 300 ms and 1:4 binning (Mediso nanoScan PET/CT, Mediso Medical Imaging Systems, Hungary) and total activity counts over 30 min were measured. PET data were reconstructed using Mediso’s iterative Tera-Tomo 3D reconstruction algorithm using 4 iterations, 6 subsets, full detector model, normal regularization, spike filter on, voxel size 0–6 mm and 400–600 keV energy window. Micro-PET-CT images were analysed on an OsiriX workstation (OsiriX version 7.5.1, 64-bit, OsiriX Imaging Software, Geneva, Switzerland). Regions of interest were drawn around contours of phantoms on the CT and mapped to corresponding fused ^18^F-fluoride positron emission tomographic images. Total binding activity curves and Scatchard plots were generated to calculate Bmax and K_d_ of ^18^F-fluoride for subsequent ex vivo experiments. To ensure saturation of binding sites, 2 × K_d_ was used to evaluate the selectivity of ^18^F-fluoride for hydroxyapatite compared with phantoms of calcium phosphate, calcium oxalate and calcium pyrophosphate using the method described above.

### Cadaveric coronary arteries and ex vivo ^18^F-fluoride micro-positron emission tomography computed tomography

Atherosclerotic sections of left coronary arteries were obtained from victims of sudden death (both cardiac and non-cardiac) with ethical approval and informed relative authorisation from the next of kin (National Health Service South East Scotland Research Ethics Committee 14/SS/1090). Tissue was independently obtained at time of autopsy by the performing pathologist (RB). Legislation regarding the regulation of tissue in victims of sudden death meant that only left main and proximal left anterior descending coronary artery specimens could be obtained for detailed research analysis. These were obtained with the surrounding myocardium and did not necessarily include the specific culprit plaque for patients who had suffered acute myocardial infarction. Tissue was immediately fresh frozen at − 80 °C. Thawed non-decalcified coronary artery specimens were incubated for 20 min in ^18^F-fluoride 100 kBq/mL solution (10.5 MBq ^18^F-fluoride in 99.5 mL 0.9% sodium chloride). Specimens were twice washed in 10 mL 0.9% sodium chloride for 5 min to remove unbound ^18^F-fluoride. Specimens were scanned using the microPET-CT protocol described above. Regions of interest were drawn in background regions, myocardium, non-calcified and calcified segments in coronary artery plaques using micro-CT images and the maximum ^18^F-fluoride activity in each region was recorded on co-registered micro-PET images. Maximum activity recorded in a region equal to or above 100 kBq/mL was defined as high-intensity ^18^F-fluoride (> 5 × myocardium activity), whereas values with a maximum activity of less than 100 kBq/mL was defined as low-intensity. After whole specimen imaging, the coronary arteries were fixed in 10% (w/v) neutral buffered formalin.

### Sample preparation and histological examination

Formalin-fixed coronary arteries were sectioned into 2–4 mm slices by a vascular biology/pathology lab (MS, SS). The resulting slices were embedded in paraffin which provided 1–8 tissue cross-sections analysed per sample depending on the size of tissue initially isolated. Paraffin sections (4 µm) were used for histology and immunohistochemistry as described below. In all cases, images were generated using an Aperio Slide Scanner using ImageScope software (Leica Biosystems, Germany). Histological examination was performed using haematoxylin and eosin staining for overall pathology, followed by Movat’s pentachrome and trichome staining to differentiate fibrosis and elastic fibres. Von Kossa and Alizarin Red S staining were used to assess for the presence of calcification.

### Fluorescein-bisphosphonate immunofluorescence of cadaveric coronary arteries

To determine whether the binding of ^18^F-fluoride in regions of Von Kossa and Alizarin Red S was specific for hydroxyapatite deposition, categorisation of these regions using a fluorescein-bisphosphonate probe was undertaken. Fluorescein-bisphosphonate is a highly sensitive and specific probe for identifying regions of microcrystalline hydroxyapatite. Incubation and binding in tissue has previously been described in detail^13^. Briefly, sections were de-waxed in xylene and incubated with fluorescein-bisphosphonate (1 µM) for 2 h, washed in water (2 ×) followed by incubation with 2% Alizarin Red S (250 µL) for 5 min. Sections were washed in water (3 ×) and subsequently incubated with 4′,6-diamidino-2-phenylindole (500 nM) for 5 min. Sections were washed with water (1 ×) and then mounted using ProLong Gold Antifade. Fluorescence signal was detected under a Leica DMRB fluorescence microscope.

### Raman spectroscopy

Coronary artery specimens were embedded in paraffin wax, sectioned and subsequently placed on calcium fluoride slides. Once dried, the slides were placed in xylene for 15 min followed by dehydration in ethanol. As soon as they were dehydrated, the sections were ready for imaging and did not require additional processing. Alizarin Red S staining was used to discriminate regions of calcification. Sections from tissue presenting no calcification, calcification with low fluoride intensity (< 100 kBq/mL), and no calcification with high fluoride intensity (≥ 100 kBq/mL) were selected in order to address whether there were any differences in the apatite crystal or molecular substitution in the structure. Raman imaging was carried out using an InVia Renishaw Microscope with a 785 nm laser excitation source which was used to excite the sample through a 50, N.A. 0.75 objective. The total data acquisition was performed during 60 s for spectra with a 100% laser power using the WiRE software (Renishaw, Gloucestershire, United Kingdom). All of the spectra acquired were background subtracted using a background correction algorithm.

### Immunohistochemical categorisation of osteogenic markers

Immunohistochemistry was completed using osteopontin (OPN) (Sigma-Aldrich Catalog No: O7264, 1:100 dilution), Runt-related transcription factor 2 (Runx-2) (Abcam Catalog No: Ab76956, 1:50 dilution), transforming growth factor beta 1 (TGFβ1) (Abcam Catalog No: Ab64715, 1:25 dilution), wingless/integrated 3a (Wnt3A) (Abcam Catalog No: Ab28472, 1:200 dilution) and Caspase 3 (Cell Signalling No: 9664, 1:100 dilution). Staining was performed via automated staining with a Leica Bond Rx system using Bond Epitope Retrieval Solution 1 (pH = 6, Catalog No: AR9961) and Bond Polymer Refine Red Detection (Catalog No: DS9390). The omission of the primary antibody served as negative controls. Blinded qualitative categorisation of nuclear staining intensity was performed by a pathologist using a 4-point classification: 0, no notable staining, 1, < 20% of plaque or relevant cells are weakly positive, 2, < 50% of plaque or some relevant cells are strongly positively, 3, 50–100% of plaque or all relevant cells are strongly positive. For comparison of immunohistochemical analysis with ^18^F-fluoride activity, plaques with a classification 3 were defined as ‘positive’ and classification < 3 was defined as ‘negative’ for osteopontin, runt-related transcription factor 2, transforming growth factor beta 1 and wingless/integrated 3a. Plaques with classification 2 were defined as ‘positive’ and classification < 2 were defined as ‘negative’ for Caspase 3.

### Statistical analysis

Categorical variables are reported as number (%) and continuous variables as mean ± standard deviation for parametric or median and interquartile range for non-parametric data. Normality was tested for using the D’Agostino and Pearson test. Continuous unpaired variables were compared using Student’s *t* test with Welch’s correction when two samples had unequal variances and/or unequal sample sizes. Non-parametric data was compared between two categories using Mann–Whitney *U* test or using Kruskal–Wallis test for multiple categories. Statistical analysis was undertaken using PRISM for OS X, version 8.1.1 (GraphPad Software, San Diego, California, USA). Statistical significance was considered as a two-sided p value < 0.05.

### Ethical approval

Tissue samples were obtained at autopsy with written informed relative authorisation from the next of kin. Ethical approval was granted by National Health Service Research Ethics Committee South East Scotland (14/SS/1090). The retention, storage and use of tissue sections were compliant with the United Kingdom Human Tissue Act of 2004 and in accordance with the relevant guidelines and regulations approved by National Health Service Research Ethics Committee South East Scotland (14/SS/1090).
